# Timing–dependent responses of *Fusarium graminearum* suppression and malting quality in barley following PSP1 elicitor application

**DOI:** 10.3389/fpls.2026.1838206

**Published:** 2026-05-12

**Authors:** Mauro Martínez, Juan Izquierdo, Adriana Arrigoni, Germán González, Björn Welin, Rodomiro Ortiz, Sebastiàn Stenglein

**Affiliations:** 1 Instituto de Biología Funcional y Biotecnología (BIOLAB–CONICET), Buenos Aires, Argentina; 2 Facultad de Agronomía (FAA–UNCPBA), Universidad Nacional del Centro de la Provincia de Buenos Aires, Buenos Aires, Argentina; 3 Group for Biotechnology and Agri–food Sustainability (ABSA), Punta del Este, Uruguay; 4 Instituto Nacional de Tecnología Agropecuaria, INTA EEA–Bordenave, Buenos Aires, Argentina; 5 Instituto de Tecnología Agroindustrial del Noroeste Argentino, Estación Experimental Agroindustrial Obispo Colombres (CONICET), Tucumán, Argentina; 6 Department of Plant Breeding, Swedish University of Agricultural Sciences, Alnarp, Sweden

**Keywords:** cereals, climate change, elicitor, fungal diseases, malt, plant–pathogen interaction

## Abstract

**Introduction:**

Plant defence elicitors have emerged as promising tools and a sustainable alternative to enhance crop resilience. In barley, the potential benefits of elicitors on agronomic performance remain insufficiently understood. The present study aimed to evaluate the effects of the defence elicitor *Plant Stimulator and Protector 1* (PSP1) on the *Fusarium graminearum*–barley pathosystem in the Argentine Pampas, considering application timing.

**Methods:**

Field experiments were conducted in 2022 and 2023 using two contrasting commercial two–row spring barley genotypes. The PSP1 was applied at three phenological stages: tillering (T1), stem elongation (T2), and heading (T3), with plots artificially inoculated with *F. graminearum (DC.55)*. Disease parameters, yield components, commercial grain traits and industrial malting quality variables were assessed.

**Results and discussion:**

The results showed that applications close to heading resulted in low, non–significant reductions in FHB incidence (≤10%) and severity (≤5%) relative to earlier applications. Grain yield components were largely unaffected by PSP1, whereas malting quality showed a clear change in response to defence activation. Late applications tended to negatively affect malt extract, friability, and the Kolbach index (≤5%), as well as FAN and filtration time (≤25%) compared to the control. In contrast, malt protein, grain size, and wort pH increased. Under low–moderate FHB pressure, PSP1 application timing was a key determinant of barley agronomic and technological outcomes, with earlier applications linked to better malting quality. These results provide novel field–based insights into elicitor use in barley, supporting the design of future multi–environment studies to optimize the deployment of elicitor–based strategies.

## 1 Introduction

Plant biostimulants include diverse substances and microorganisms that enhance plant growth, such as humic and fulvic acids, microbial inoculants, proteins and amino acids, and seed or plant extracts, among others (Brown and Saa, 2015; Calvo et al., 2014; Silvestro et al., 2025). Although the use of biostimulants in agricultural systems has increased significantly over the last few decades, a clearer understanding of their effects, particularly under field conditions, remains needed (Calvo et al., 2014; Brown and Saa, 2015; Flavell et al., 2025). According to the European Biostimulants Industry Council, biostimulants are products that enhance plant nutrient use, stress tolerance, and productivity by stimulating natural physiological processes rather than supplying nutrients directly (EBIC, 2025). Among these novel molecules, elicitors appear to be a powerful tool for enhancing plant resilience to abiotic and biotic stress (Abdul Malik et al., 2020). Elicitors can be natural or synthetic compounds that induce defence responses similar to those triggered by pathogen infection, herbivory, and other stresses (Thakur and Sohal, 2013). Currently, different types of elicitors have been characterized and are commercially available, including carbohydrate, glycopeptides, glycoproteins, lipids, and polymers (Garde–Cerdán et al., 2022).

One of the most promising commercial elicitors is PSP1 (*Plant Stimulator and Protector 1*), a biopesticide formulated based on an extracellular fungal protein. This extracellular protease subtilisin (AsES) was obtained from *Sarocladium strictum* (strain SS71), a fungal species associated with strawberry infections (Chalfoun et al., 2013). The effect of this elicitor is based on the triggering of an oxidative burst (ROS and NO production), phenolic accumulation, a localized hypersensitive response, necrotic cell death, cell wall reinforcement through lignification and callose deposition, salicylic acid accumulation, and activation of systemic acquired resistance (SAR)–related defence genes (e.g., PRs) (Chalfoun, 2009). The benefits of PSP1 have been well documented in several agronomic crops, including strawberry and soybean, and have been reported to have positive effects mainly against fungal pathogens (*Cercospora*, *Colletotrichum*, and *Septoria*) (Chalfoun et al., 2018). However, it remains unclear how this bioproduct can protect other crops under field conditions and what its potential impact on other agronomic traits might be.

Barley (*Hordeum vulgare* L.), the fourth most extensively cultivated cereal worldwide, occupies around 47 million hectares worldwide and is mainly produced for livestock feed (70–85%), malting (15–20%), and seed production (5%) (FAOSTAT, 2025). This crop is particularly cultivated in regions characterized by short growing seasons, low rainfall, and high evaporative demand, where its adaptability ensures reliable yields compared to other crops (Miralles et al., 2020). Usually, barley grain yield and malting quality are influenced by a range of biotic and abiotic stresses that may arise at different stages of the crop cycle, frequently interacting and co–occurring (Martínez et al., 2025). Among these factors, fungal diseases become more relevant under rainfed conditions and in the context of climate change (CC) scenarios (Gautam et al., 2013). Foliar diseases such as net blotch (*Dreschlera* spp.), scald (*Rynchosporium*), and spot blotch (*Bipolaris*) take the primary focus in barley due to their negative impact on the foliar area and subsequently on grain yield (Carmona et al., 2011; Walters et al., 2012). However, other secondary diseases in barley become relevant under zero–tillage systems, such as Fusarium Head Blight (FHB), caused by several species of the *Fusarium* genus (Salas et al., 1999; Sarlin et al., 2005). Among the most relevant species, *F. graminearum* and *F. poae* are among the most frequently isolated from barley grains in several agricultural systems (Nogueira et al., 2018; Alaniz–Zanon et al., 2025). In Argentina and Uruguay, the predominant species in barley grains have been *Fusarium graminearum* and *F. poae*, producing harmful mycotoxins such as deoxynivalenol (DON) and nivalenol (NIV), respectively (Nogueira, 2020). The European Union has established a maximum limit of 1.25 µg/g^-1^ of DON in unprocessed grains, while a maximum limit for NIV has not yet been established, although NIV is more toxic than DON (Nagashima, 2018). These toxins can not only be present in unprocessed grains but are also stable after common food and beverage processing (Manno et al., 2024; Martínez et al., 2020; Nogueira et al., 2018). Although the impact of FHB on barley grain yield is not as pronounced as in wheat, the adverse effects on malting parameters are relevant for food safety (Nielsen et al., 2014; Stenglein et al., 2014).

Historically, the FHB management has relied on agronomic strategies such as tillage practices, the selection of resistant cultivars (e.g., FHB1 and FHB2 quantitative trait loci –QTLs), appropriate crop rotation (avoiding cereals as preceding crops), efficient weed management (reducing alternative hosts), and the application of triazole–based fungicides during wheat anthesis (Beyer et al., 2006; Brar et al., 2019). Thus, chemical control remains one of the most frequently employed measures to prevent and mitigate FHB infection, although its effectiveness under field conditions has been questioned. Several studies have shown that triazole fungicides, such as cyproconazole, prothioconazole, and tebuconazole, as well as other azole molecules, can substantially reduce disease incidence and severity (Duan et al., 2018; Paul et al., 2007). Conversely, strobilurin fungicides (e.g., azoxystrobin) may offer only partial FHB suppression, whereas other molecules, such as carbendazim and prochloraz, have demonstrated potential (when combined with triazoles or strobilurins) to enhance control efficacy and delay the development of pathogen resistance (Duan et al., 2019; Haidukowski et al., 2005; Vogelgsang et al., 2019).

Several studies indicate that, over the coming decades, the use of agrochemicals should be limited or reduced, mainly due to their limited effectiveness against several pathogens and reports of new resistance (Hahn, 2014; Ishi and Holloman, 2015; Yin et al., 2023). The use of fossil fuels, land degradation, biodiversity loss, and excessive use of agricultural inputs (e.g., fertilizers and agrochemicals), among other factors, have increased, negatively impacting human and animal health and biodiversity (Lee et al., 2023). Under CC scenarios, fungal disease pressure is expected to increase in agricultural systems worldwide, with FHB not being an exception (Elad and Pertot, 2014; Gautam et al., 2013; Paterson et al., 2013). In this way, plant defence elicitors emerge as a valuable alternative to complement, or even replace, these synthetic molecules, thereby reducing environmental impact (Calvo et al., 2014). In previous studies, we evaluated the effect of PSP1 on wheat crops under field conditions, revealing novel insights into its use in rainfed conditions (Martínez et al., 2024). In barley, promising results for yield and profitability were obtained in on–farm barley tests when a humic biostimulant was foliar–applied once at Z1.6 (Izquierdo et al., 2025). However, scarce information is available on how elicitor applications could modulate plant–pathogen interaction in this crop under field conditions. Thus, the objective of the present work was to evaluate the potential impact of PSP1 on the *F. graminearum*–barley pathosystem, explicitly considering application timing across phenological stages. More specifically, the effects on disease parameters, grain yield, and commercial and malting quality were explored in the present work.

## 2 Materials and methods

### 2.1 Field experiments

Experiments were conducted over two consecutive growing seasons (2022/2023 and 2023/2024) under field conditions. The chosen experimental site was the UNCPBA–FAA experimental farm (Azul, Buenos Aires, Argentina–36°49’41.40” S, 59°53’11.60” W). The soil was a typical Argiudoll, well–drained and free of impediments to root development, with the following composition at 0–20 cm: clay loam texture, 5.47 kg N ha^-1^ (reflectometry), 14.15 ppm of available phosphorus (Bray–Kurtz I method), 4.21% organic matter (Walkley–Black method) and a pH of 6.77 (1: 2.5 in water).

For the present study, two representative spring barley genotypes (two–row) were chosen, considering their similar crop cycle length (short–intermediate): cv. Andreia and cv. Overture (Cattaneo and Cortese, 2025). The genotype cv. Andreia (ABInbev^®^, Belgium, 2011) is a commonly used cultivar in the Argentine pampas over the last decade, with moderate grain yield potential and good malting performance (protein content and screening percentage), but is susceptible to several foliar diseases. Meanwhile, the genotype cv. Overture (Cerfoly^®^, Argentina, 2016) is a modern cultivar with moderate grain yield potential and stable malting performance, with tolerance to several foliar diseases (Cattáneo and Cortese, 2025). Regarding FHB behaviour, no prior screening data are available for either genotype, both of which are considered moderately susceptible. Moreover, both genotypes play a key role, being responsible for around 50% of the barley sown area in the study region, with 38% (400–000 ha) for cv. Andreia and 16% (200–000 ha) for cv. Overture during the 2024/2025 growing season (INASE, 2025).

The crop rotation system was wheat–fallow, with alternating crops such as barley, peas, and quinoa. The chosen tillage practice was a zero–tillage system. Sowing was conducted on 15th July for both growing seasons (hereafter, 2022 and 2023) to avoid shifts in phenological phases and ensure uniformity in heading dates and treatment applications. Each plot size was 10 m x 1.4 m (seven rows at 0.21 cm) with a plant density of 250 seed m^-2^. Field experiments were conducted under rainfed conditions, without supplementary irrigation. Nitrogen was supplied at a rate of 150 kg N ha^-1^ as urea (46–0–0), applied in two split doses: 40% at sowing and 60% during the tillering stage. Weed management was achieved by applying conventional herbicides (glyphosate, metsulfuron–methyl, and dicamba) at recommended rates, while no insecticide or fungicide treatments were applied throughout the crop cycle.

### 2.2 PSP1 application and *Fusarium* treatment

To evaluate the potential effect of PSP1 on FHB development under field conditions, the experimental design was a split–plot in a randomized complete block design (RCBD) with three replicates. The two commercial spring barley genotypes (cv. Andreia and cv. Overture) were assigned to the main plots, and each main plot was randomly divided into four subplots, each assigned to one of four elicitor treatments (T, T1, T2, and T3). Each experimental block consisted of two primary plots (10 m × 1.4 m), each containing both barley genotypes. These plots were further divided into four subplots (2.5 m × 1.4 m) per genotype, to which the different elicitor treatments were randomly assigned. The commercial formulation PSP1 (Howler^®^, Sumitomo Chemical, Japan) was used across all treated subplots. Applications were prepared according to the manufacturer’s recommendation for barley (2 L ha^-1^); whereas an adjuvant composed of ethoxylated fatty amines (0.5%) was included as a cationic surfactant to enhance foliar absorption of the product. The PSP1 solution was applied using a Giber^®^ (Buenos Aires, Argentina) 5 L manual sprayer equipped with a flat–fan nozzle (2 bar), delivering approximately 100 L ha^-1^. Three application timings were evaluated: (i) early treatment (T1) at tillering stage (DC.21); (ii) intermediate treatment (T2) at stem elongation stage (DC.31); and (iii) late treatment 3 (T3) when 50% of plants had reached heading stage (DC.55), 48 h before fungal inoculation (Schöneberg et al., 2018; Zadoks et al., 1974). Control plots (T) received only sterile distilled water.

Regarding *Fusarium* inoculation, *F. graminearum sensu stricto (s.s.)* isolates were employed, as this species is among the most frequently isolated from barley grains in the study region (Nogueira et al., 2018). Four *F. graminearum* isolates (No. 3.4, 88.1, 92.2, and 129.1) were previously obtained from a field survey of barley crops in the study region in the Argentine Pampas region (2010–2012) (Castañares et al., 2014). Morphological and molecular identification was carried out, as well as the measurement of fungal aggressiveness and mycotoxin contamination (DON, 3–ADON, and 15–ADON production) (Castañares et al., 2014; Martínez et al., 2020). Each isolate was grown on 2% potato dextrose agar (PDA) plates for seven days at 25 ± 2 °C under a 12 h photoperiod. Macroconidia were produced by transferring five mycelial plugs (1 cm²) from actively growing colonies into 50 mL of carboxymethylcellulose (CMC) broth contained in 250 mL Erlenmeyer flasks. The CMC medium consisted of 15 g CMC, 1 g NH_4_NO_3_, 1 g KH_2_PO_4_, 1 g MgSO_4_·7H_2_O, 1 g yeast extract, and 1 L of sterile distilled water. Cultures were incubated in darkness on a rotary shaker at 100 rpm and 25 ± 2 °C for 10 days. After incubation, the suspensions were filtered through cheesecloth, and spore concentrations were adjusted to 1 × 10^5^ conidia mL^-1^ using a Neubauer hemocytometer. Equal volumes of each isolate were then combined to form the final inoculum, to which 0.05% Tween 20 was added as a surfactant. In all growing seasons, both genotypes reached 50% heading stage (DC.55) simultaneously in late October (around 25^th^ October). At this stage, barley spikes (awns) were sprayed to run–off with 250 mL of the conidial suspension per subplot using a 2 L handheld sprayer equipped with adjustable brass nozzles. Applications were preferably conducted in the late afternoon on cloudy days with relative humidity exceeding 80%, ensuring favourable conditions for pathogen infection and disease establishment. Due to heterogeneity in barley heading among genotypes, a reinoculation was performed 48 h after the first inoculation to ensure inoculum availability for all plants evaluated.

Finally, climatic conditions (rainfall and temperature) were monitored from inoculation to harvest using data loggers placed at spike height in each treatment (Cavadevices^®^, Buenos Aires, Argentina). To assess interannual variability, climatic anomalies were identified with the Giovanni NASA–EarthData system (v.4.40) by comparing each growing season (2022 and 2023) with the 20–year historical average (2003–2023) ± two standard deviations (Acker and Leptoukh, 2007; Guttman, 1999; Hansen et al., 2012). Monthly rainfalls data were obtained from the GPM–IMERG dataset, and mean temperatures from MERRA–2 (GMAO, 2015; Huffman et al., 2019). Furthermore, the Oceanic Niño Index was used to characterize ENSO phases (ONI and National Weather Service, Climate Prediction Center, 2024).

### 2.3 Measurements

A total of 21 agronomic variables were evaluated over the two growing seasons, grouped into five categories: disease variables, grain yield, commercial parameters, and industrial parameters. Regarding disease variables, disease incidence was evaluated after inoculation (21 days post–inoculation–dpi) by counting the number of symptomatic spikelets for *F. graminearum s.s.*, identifying bleaching and typical FHB lesions. Forty spikes per subplot were selected randomly, according to Campbell and Lipps (1998). The disease incidence (DI) was calculated as the percentage of symptomatic spikes divided by the total number of spikes evaluated per subplot, whereas disease severity (DS) was calculated as the ratio of symptomatic spikelets to the total number of spikelets per spike. After reaching physiological maturity, the plots were manually harvested, with the two outer rows removed. Spikes were collected exclusively from the five central rows within a 1 m × 1 m area and threshed using a stationary cereal thresher (Forti^®^, Buenos Aires, Argentina). The resulting grain samples were then homogenized using a grain divider (CerealTools^®^, Rosario, Argentina) to obtain a representative subsample, and grain yield m^-^² (GY) was determined. Moreover, in each grain subsample, discoloured, shrivelled, and chalky kernels were identified and quantified as *Fusarium*–damaged kernels (FDK). Hectolitre weight (HW), thousand kernel weight (TKW), protein content (PC), and grain moisture were measured using a near–infrared transmission spectroscope equipped with a double–face monochromator (NIT Agricheck^®^, Bruins Instruments, USA).

After NIT measurements, other barley commercial parameters, such as germinative power (GP), screening percentage (SP), and water sensitivity (WS), were also calculated. The GP was registered after seven days, according to ISTA protocols (ISTA, 2017). To determine the SP, barley grains were sieved for 5 minutes, and the proportion of grains retained on a 2.5 mm sieve was defined as the screening percentage (SP2.5), whereas the fraction passing through a 2.2 mm sieve was also recorded (SP2.2). Regarding WS, this parameter was assessed using the Pollock test (Arias, 1991; Espasandín Bartesaghi, 2010). For each sample, two replicates of 100 grains were placed in 90 mm Petri dishes, one containing 4 mL of sterile distilled water to represent normal conditions, and the other containing 8 mL to simulate water excess. After 72 hours, grains with radicles exceeding 2 mm were counted, and the difference between treatments was expressed as WS. Moreover, to evaluate seed health, blotter tests with deep–freezing (BTDF) assays were conducted, and results were reported as the percentage of healthy grains (Camargo et al., 2017).

For the micromalting analysis, barley grains were stored for two months in plastic containers under dry conditions at 18 °C to overcome post–harvest dormancy (Habschied et al., 2024). Before malting, each sample was cleaned, conditioned, and size–graded using a grain classifier (Lobofix^®^, Buenos Aires, Argentina). A 250 g portion of each sample was micromalted in a replicate of a Phoenix Automated Micromalting System (Phoenix Byosistems^®^, Adelaide, Australia), following a process that included 24 h of steeping at 15 °C, 96 h of germination (air rest) at 15 °C, and 24 h of stepped kilning (50–82 °C). The resulting malt was analyzed for quality traits according to European Brewery Convention (EBC, 1987) methods, being the following variables evaluated: friability (FB), malt extract (ME), saccharification time (ST), filtration time (FT), wort pH (WpH), malt total protein (MP), soluble protein (PS), Kolbach index (KI), and free amino nitrogen (FAN). All the previous measurements were performed in duplicate.

### 2.4 Statistical analysis

Given the contrasting climatic conditions between the two years evaluated, the datasets from 2022 and 2023 were analyzed independently. All statistical procedures were performed using RStudio v.4.5.0 (R Core Team, 2025). For each experimental year, the main factor was the barley genotype (*cv.* Andreia and cv. Overture), whereas treatments (T, T1, T2, and T3) were nested within subplots, with three replicated plots (blocks) for each treatment–genotype combination. Data were analyzed using mixed–effects models that accounted for the hierarchical structure of the experiment by nesting plots within blocks and subplots within plots. Model specification and selection were based on the Akaike Information Criterion (AIC), retaining the model with the best fit that included genotype (G) and treatment (T) as fixed factors, and block as a random factor. Model assumptions were verified using Q–Q plots, the Shapiro–Wilk test for normality, and Levene’s test for homogeneity of variance. For response variables following a normal error distribution (GN, GW, GY, HW, ME, MP, PC, PS, KI, FAN, WS, and WpH), analyses were carried out using the lmer function (linear mixed–effects model) from the lme4 package (Bates et al., 2015). Categorical traits (FT and ST) were converted to continuous scales to approximate normality, allowing their inclusion in a linear mixed–effects modelling framework. Variables exhibiting non–normal error structures (count or proportion data) were analyzed using the glmer function (generalized linear mixed–effects model) from the lme4 package, specifying a binomial family with a logit link (BTDF, DI, DS, FDK, GP, MF, SP2.5, and SP2.2). Furthermore, application timing was structured as an ordinal factor with three predefined phenological stages representing crop development, and linear and mixed–effects regression models were used.

Analysis of variance (ANOVA) was performed for each variable, and significant effects were further examined using the lsmeans function from the emmeans package (Lenth, 2018). Pairwise comparisons were carried out with Tukey’s *post hoc* test (α = 0.05), and data were presented as estimated marginal means (EMMs) with 95% confidence intervals, whereas figures displayed raw means ± standard error of the mean (SEM). Additionally, Principal Component Analyses (PCA) were conducted independently for each year to identify patterns of multivariate variability, with scree plots used to determine the number of principal components retained. Spearman’s rank correlations (r_s_) were computed to explore monotonic associations among variables with non–normal distributions, whereas simple linear regressions were used to describe directional relationships between the timing of elicitor application and response variables.

## 3 Results

### 3.1 Climatic conditions and disease parameters

**Figure 1:**
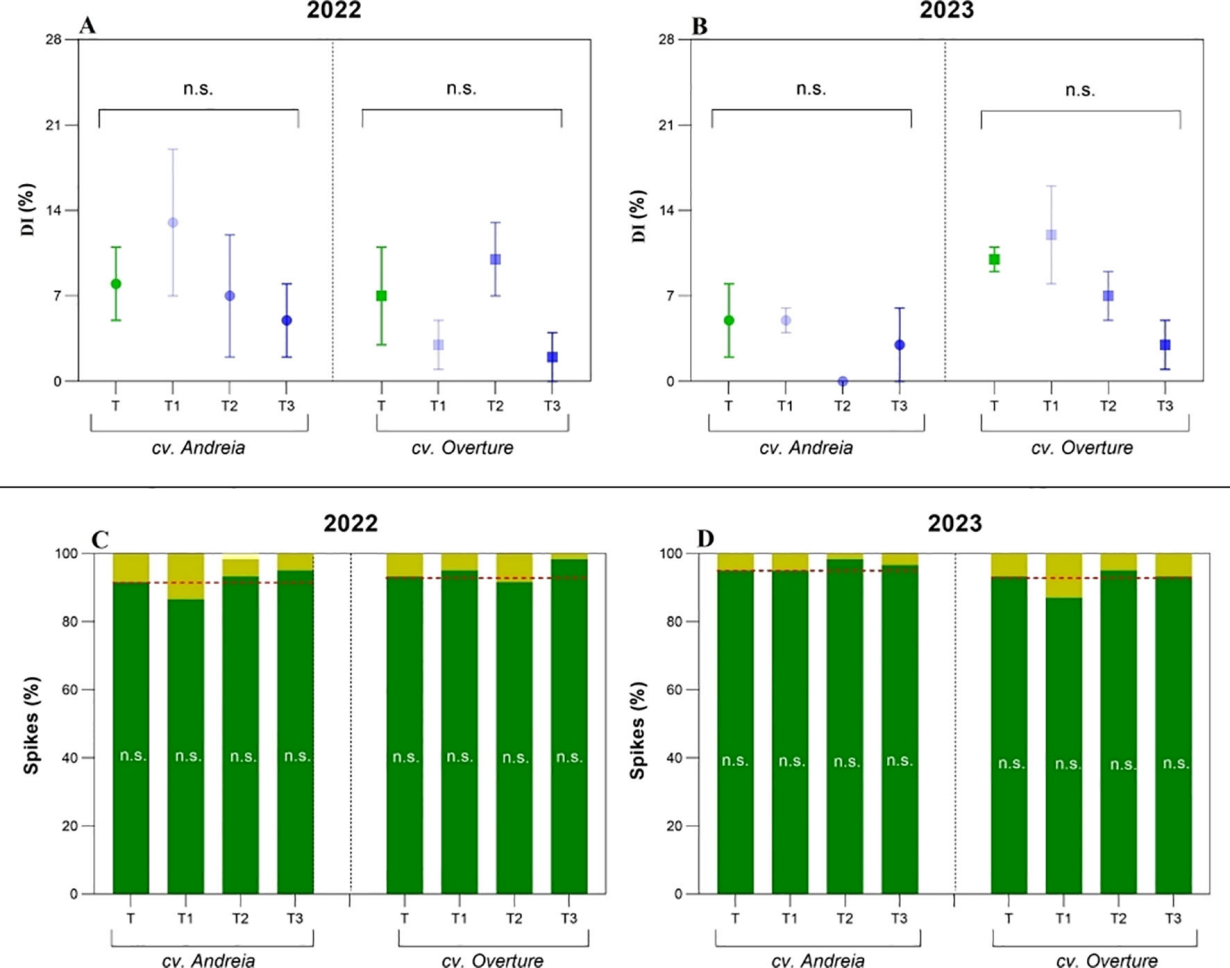
Disease incidence (DI, %) and severity (DS, % spikes) partitioned by year (2022 and 2023) and genotypes (cv. Andreia and cv. Overture). **(A)** DI in 2022. **(B)** DI in 2023. **(C)** DS in 2022. **(D)** DS in 2023. Treatments: PSP1 applied at the tillering stage (DC.21, T1); at the stem elongation stage (DC.31, T2); at 50% of heading (DC.55, T3); and control treatment (T).

Climatic conditions were contrasting during the years evaluated (2022 and 2023). Regarding the ONI index, the year 2022 was characterized as “La Niña” ENSO phase (from -0.8 °C to -1 °C), with drier and higher temperatures, whereas the year 2023 was characterized as “El Niño” ENSO phase (0.8 °C to 2.0 °C), with moderate temperatures and humid conditions for the study region. For the FHB susceptibility period (November) in 2022, we recorded temperatures above the historical average (+11.8%, 19.5 °C) and rainfall around the historical average for this month (+6.7%, 111 mm). In contrast, during the FHB susceptibility period (November) in 2023, we recorded temperatures around the historical average (-0.6%, 17.3 °C) and rainfall above the historical average for this month (+46.3%, 152.5 mm). Furthermore, during grain filling (December) in 2023, a rainfall anomaly was observed, with rainfall 142.55% above the historical average (227.7 mm). All these changes described in environmental conditions during the FHB susceptibility period shaped disease parameters, with higher DI values in 2022 and higher DS in 2023 (**Figure 1**).

For all the disease parameters evaluated (DI, DS, FDK, and BTDF), no significant differences were observed among treatments or genotypes across the years evaluated, despite the high variability observed among treatments (**Figure 1**, **Table 1**). Considering both years, FHB symptoms were moderate to low, with the DI and DS maximum values not exceeding ~20% and 10%, respectively. For DI, we observed higher average values in 2022 than in 2023, indicating that the cv. Andreia had higher values (7.62% ± 2.1%) compared to cv. Overture (4.29% ± 1.6%), where late treatment showed the lowest DI values (2.8% ± 1.65%) (**Figure 1A**, **Supplementary Table 1**). In 2023, cv. Overture showed the highest average DI value (7.2% ± 1.78%) compared to cv. Andreia, with the intermediate treatment showing the lowest DI values (**Figure 1B**, **Supplementary Table 1**). Regarding DS, in 2022 cv. Andreia showed a higher value (4.36% ± 1.23%) than cv. Overture (0%), where the late treatment showed the lowest DS values (0%) (**Figure 1C**, **Supplementary Table 1**). In 2023, cv. Overture had the highest DS value (3.27% ± 1.14%) compared to cv. Andreia (0%), with intermediate (0%) and late treatments (1.39% ± 0.98%) showing the lowest DS values (**Figure 1D**, **Supplementary Table 1**). Regarding FDK, the symptomatic grains recorded were scarce, with the highest FDK values reported in 2022 (0.20%–0.35%), whereas for asymptomatic grains, the highest BTDF values were similar across years, genotypes, and treatments, ranging from 0.81% to 2.50% (**Supplementary Table 1**).

**Table 1:** Analysis of variance (ANOVA) for the disease parameters evaluated.

Y	S.V.	d.f.	DI	DS	FDK	BTDF
*2022*	T	3	0.4003	0.8353	0.4456	0.8549
	G	1	0.7280	0.6511	0.6375	0.7375
	T * G	3	0.2761	0.9223	0.9000	0.9919
*2023*	T	3	0.9677	1.0000	0.1143	0.1660
	G	1	0.3075	0.3341	0.9745	0.4805
	T * G	3	0.9039	0.8357	1.0000	0.6991

DI, disease incidence; DS, disease severity; FDK, *Fusarium* damaged kernel; BTDF, blotter test deep freezing.

Y, year; S.V., source of variation; d.f., degree of freedom. *Tukey test (α=0.05). n.d., not detected.

### 3.2 Grain yield parameters

**Figure 2:**
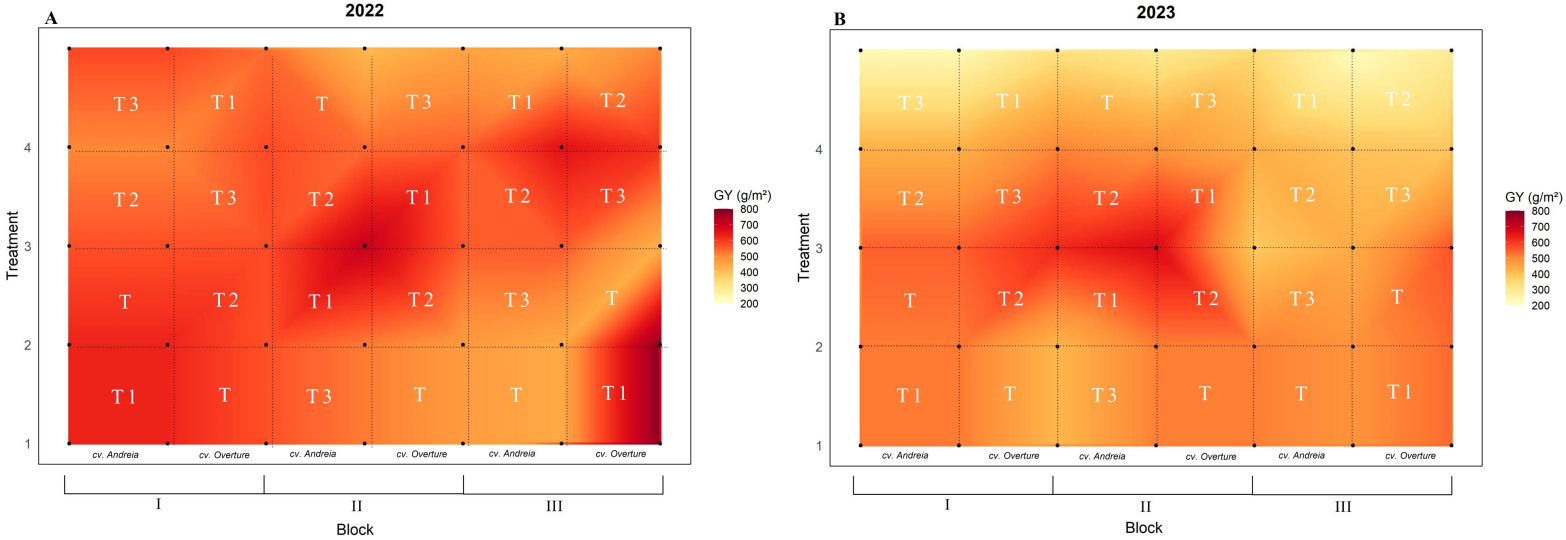
Grain yield (GY, g m^-2^) partitioned by year (2022 and 2023) and genotypes (cv. Andreia and cv. Overture). **(A)** GY in 2022. **(B)** GY in 2023.

No significant differences were detected for the grain yield parameters evaluated (GN, GW, GY, and HW) (**Table 2**). For GN, in 2022, both genotypes showed similar values (~11–500 grains m^-2^), with the highest value observed for the early treatment (12 656 ± 788 grains m^-2^), whereas in 2023 the values were similar between the two genotypes and among treatments, with the lowest GN value recorded for the late treatment (8 315± 1–122 grains m^-2^) (**Supplementary Table 2**). As for GW, this parameter was stable across years, genotypes, and treatments, being only slightly affected and ranging from 47.3 g to 48.65 g (**Supplementary Table 2**). Regarding GY, in 2022, both genotypes showed similar GY values (~550 g m^-2^), where early treatment showed the highest GY (604.08 ± 36.59) (**Figure 2A**, **Supplementary Table 2**). In 2023, the GY was lower, around 440 g m^-2^ for both genotypes, with the late treatment showing the lowest GY (407.28 ± 54.61) (**Figure 2B**, **Supplementary Table 2**). As to HW, values were similar across years, genotypes and treatments, ranging from 61.93 kg hl^-1^ to 65.10 kg hl^-1^, with cv. Andreia showed the highest HW values in both years (significant in 2023, p = 0.0232), whereas the intermediate (2022) and late (2023) treatments showed a slight increase (**Table 2**).

**Table 2:** Analysis of variance (ANOVA) of the grain yield parameters evaluated.

Y	S.V.	d.f.	GW	GN	GY	HW
*2022*	T	3	0.5687	0.4906	0.4793	0.4046
	G	1	0.2487	0.9706	0.8412	0.1882
	T * G	3	0.2461	0.8973	0.7415	0.1936
*2023*	T	3	0.9619	0.8876	0.9155	0.8227
	G	1	0.9594	0.5845	0.6232	0.0232^*^
	T * G	3	0.5470	0.9274	0.9423	0.7532

GW, grain weight; GN, grain number; GY, grain yield; HW, hectoliter weight.

Y, year; S.V., source of variation; d.f., degree of freedom. *Tukey test (α=0.05). n.d., not detected.

### 3.3 Commercial grain quality parameters

**Figure 3:**
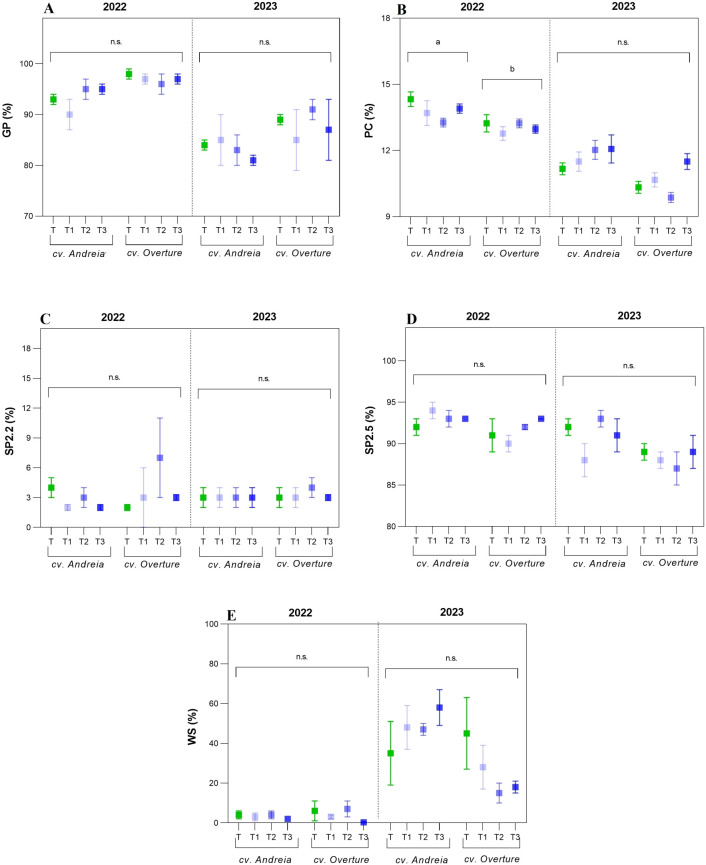
Commercial barley parameters partitioned by year (2022 and 2023) and genotypes (cv. Andreia and cv. Overture). **(A)** Germinative power (GP, %). **(B)** Protein content (PC, %). **(C)** Screening percentage < 2.2 mm (SP2.2, %). **(D)** Screening percentage > 2.5 mm (SP2.5, %). **(E)** Water sensitivity (WS, %).

Regarding commercial malting parameters (GP, PC, SP2.2, SP2.5, and WS), only PC showed a significant effect (p = 0.0046) across the genotypes evaluated (**Table 3**). Regarding GP, in 2022, no differences were observed across genotypes and treatments, with the T treatment showing the highest levels (86.19% ± 1.34%); whereas in 2023, GP values were lower, ranging from 61.58% to 64.15%, with the T treatment showing the highest values (**Figure 3A**, **Supplementary Table 3**). As for PC, significant differences were reported between genotypes in 2022, demonstrating that cv. Andreia had higher PC values than cv. Overture (13.80% vs. 13.05%) (**Figure 3B**, **Supplementary Table 3**). However, in 2023, no significant differences were detected, showing similar PC across genotypes and treatments, ranging from 10.59% to 11.58% (**Supplementary Table 3**).

**Table 3:** Analysis of variance (ANOVA) of the commercial parameters evaluated.

Y	S.V.	d.f.	GP	PC	SP2.2	SP2.5	WS
*2022*	T	3	0.7724	0.0503	0.5861	0.7897	0.9267
	G	1	0.4162	0.0046^*^	0.2493	0.7717	0.6022
	T * G	3	0.5737	0.1985	0.1533	0.6811	0.8445
*2023*	T	3	0.8079	0.2465	1.0000	0.1748	0.4962
	G	1	0.5963	0.1121	0.8157	0.1691	0.4880
	T * G	3	0.6598	0.1279	0.9920	0.2577	0.0968

GP, germinative power; PC, protein content; SP2.2, screening percentage (< 2.2 mm); SP2.5, screening percentage (>2.5 mm); WS, water sensitivity.

Y, year; S.V., source of variation; d.f., degree of freedom. *Tukey test (α=0.05). n.d., not detected.

Regarding SP2.2, during both years, cv. Overture showed a higher proportion of thin grains (3.59% and 3.33%, respectively), whereas the intermediate treatment reported the highest SP2.2 values, with 4.60% in 2022 and 3.32% in 2023 (**Figure 3C**, **Supplementary Table 3**). In the case of SP2.5, cv. Andreia showed the highest number of bold grains in both years (92.87% and 91.25%, respectively) (**Figure 3D**). Regarding the treatments, although no significant differences were detected, results indicated that in 2022 all treatments slightly increased SP2.5 relative to T, whereas in 2023 the response was erratic (**Supplementary Table 3**). As to WS, in 2022, similar values were reported across treatments and genotypes, ranging from 1.33% to 5.50%. In 2023, although no significant differences were detected, lower WS values were detected in all treatments compared to T (**Supplementary Table 3**). Although the mean ± SEM plot (**Figure 3E**) suggests genotype–specific responses in 2023, no significant genotype × treatment interaction was detected, suggesting that the observed divergence is most likely attributable to high within–group variability.

### 3.4 Malting and industrial quality parameters

**Figure 4:**
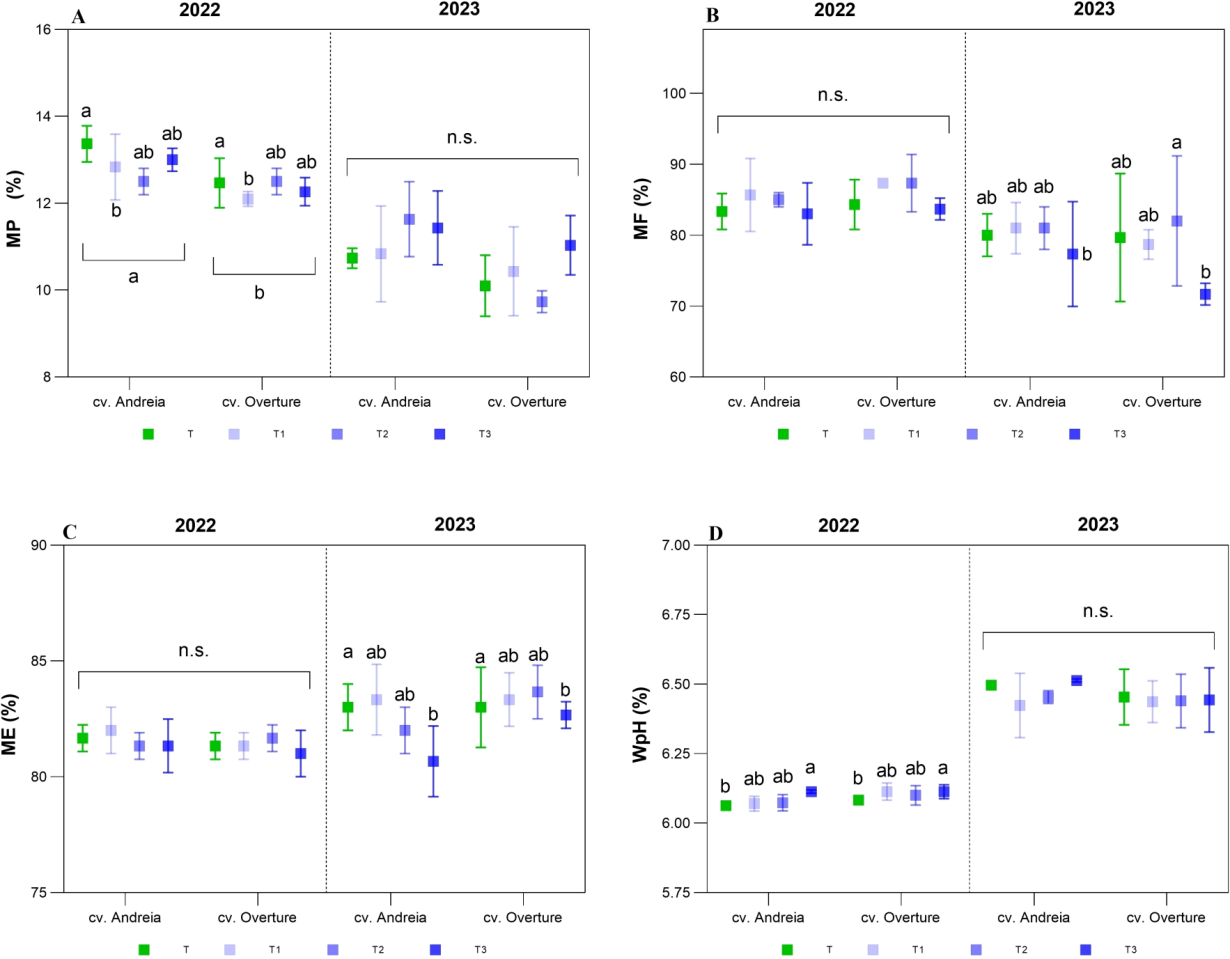
Industrial malting parameters partitioned by year (2022 and 2023) and genotypes (cv. Andreia and cv. Overture). **(A)** Malt protein (MP, %). **(B)** Malt friability (MF, %). **(C)** Malt extract (MF, %). **(D)** Wort pH (WpH, pH).

Regarding industrial malting parameters, significant effects were observed each year, except for FAN and ST (**Table 4**). For MP, in 2022, the genotype cv. Andreia showed the highest MP value (12.92% ± 0.15%; p = 0.0041); interestingly, all treatments reduced MP values (p = 0.0476), with early treatment showing the greatest impact on both genotypes (**Table 1**). However, in 2023, no significant changes were observed across genotypes and treatments, whereas cv. Andreia showed the highest MP value (11.16% ± 0.30%), with the late treatment recording the highest value (11.23% ± 0.36%) (**Figure 4A**, **Supplementary Table 4**). Regarding MF, no significant changes were observed in 2022, with the genotype cv. Overture showing the highest MF value (85.75% ± 1.01%), with early and intermediate treatments having the highest MF values (**Supplementary Table 4**). In contrast, in 2023, a significant interaction between genotype and treatment was reported (p = 0.0474), with the higher MF values recorded in cv. Overture with intermediate treatment showing the highest values. (**Figure 4B**, **Table 4**). As to ME, in 2022, no significant changes were reported, with similar values across genotypes and treatments, ranging from 81.17% to 81.77% (**Supplementary Table 4**). In contrast, in 2023, significant effects were detected only among treatments (p = 0.0170), with the highest ME in T (83.00% ± 0.56%) and the lowest in late treatment (81.67% ± 0.56%) (**Figure 4C**; **Table 1**). Regarding WpH, no significant differences were reported between the genotypes, but significant differences (p = 0.0385) were detected among treatments in 2022, with late treatment showing the highest WpH values (6.11 ± 0.01) (**Figure 4D**; **Table 1**). In 2023, no significant effects were detected across genotypes and treatments, with higher WpH values compared to 2022 (ranging from 6.43 to 6.48) (**Supplementary Table 4**).

**Table 4:** Analysis of variance (ANOVA) of the malt parameters evaluated.

Y	S.V.	d.f.	MP	MF	ME	WpH	KI	FAN	ST	FT
*2022*	T	3	0.0476^*^	0.7717	0.0667^*^	0.0385^*^	0.0573	0.5692	1.0000	8.137^-5*^
	G	1	**0.0041**	0.7394	0.2482	0.2955	0.0047^*^	0.6688	1.0000	0.0033*
	T * G	3	0.1767	0.9678	0.1337	0.4510	0.1786	0.9569	0.3916	0.0130*
*2023*	T	3	0.2653	0.2620	0.0170^*^	0.1481	0.2258	0.3065	0.3916	0.1328
	G	1	0.2447	0.9187	1.0000	0.3180	0.0593	0.9282	0.1573	0.0179^*^
	T * G	3	0.1549	0.0474^*^	0.2569	0.5554	0.0475^*^	0.1031	0.2615	0.4235

MF, malt friability; ME, malt extract; WpH, wort pH; KI, Kolbach index; FAN, free amino nitrogen; ST, saccharification time; FT; filtration time.

Y, year; S.V., source of variation; d.f., degree of freedom. *Tukey test (α=0.05). n.d., not detected.

**Figure 5:**
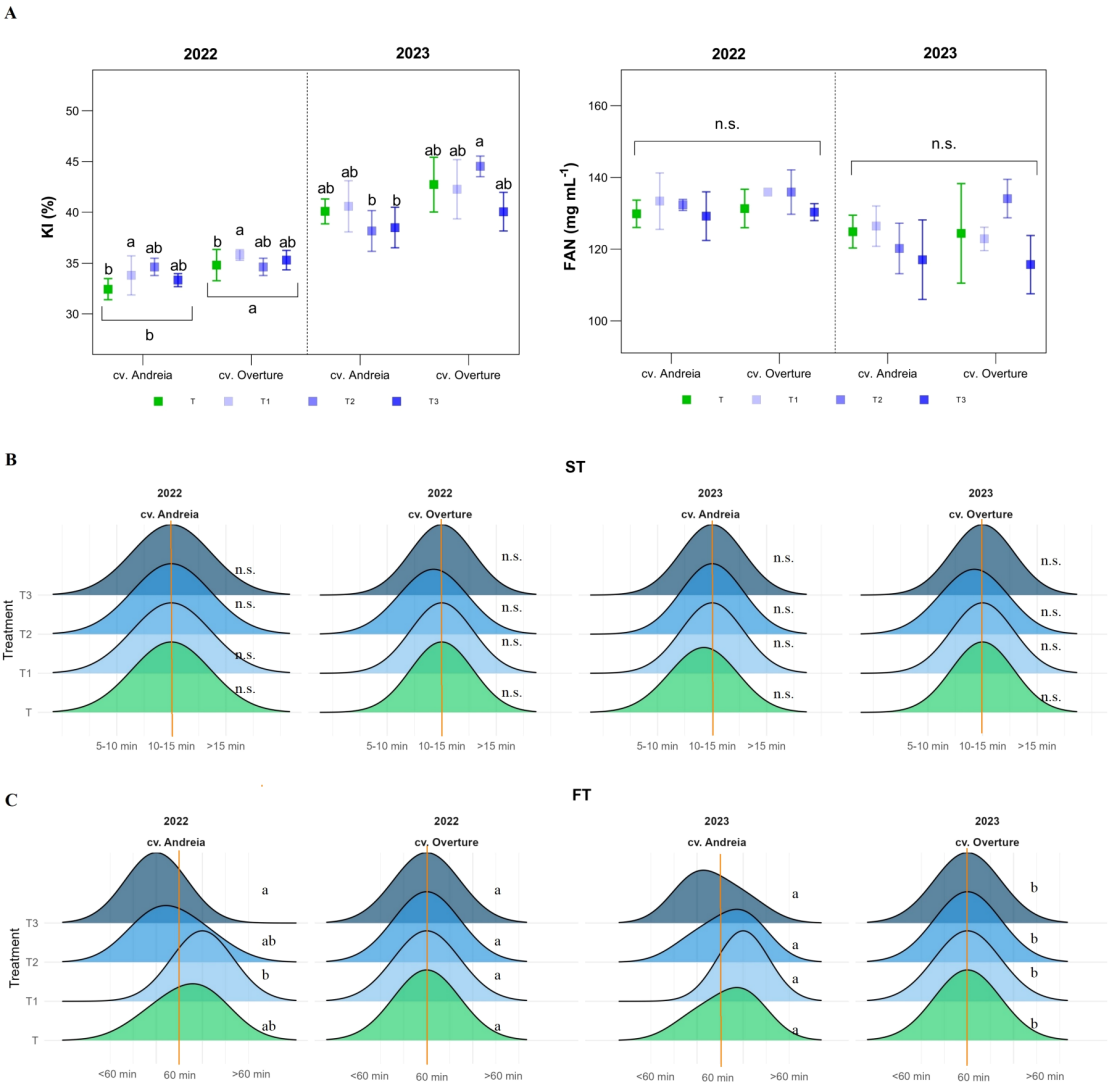
Other industrial parameters partitioned by year (2022 and 2023) and genotypes (cv. Andreia and cv. Overture). **(A)** Left: Kolbach Index (KI, %). Right: Free amino–nitrogen (FAN, mg L^-1^). **(B)** Saccharification time (ST, min). Left: 2022. Right: 2023. **(C)**. Filtration time (FT, min). Left: 2022. Right: 2023.

Regarding KI, in 2022, significant differences were reported across genotypes (p = 0.0047) (**Table 4**). The genotype cv. Overture showed higher KI values (35.13%), with early treatment reporting the highest values (34.80%) (**Figure 5A**, **Supplementary Table 4**). In 2023, an interaction between genotypes and treatments was detected (p = 0.0475), with higher KI values in cv. Overture (intermediate), whereas lower KI values were registered in cv. Andreia (intermediate and late treatments) (**Figure 5A**). As for FAN values, no significant differences were observed between the two years, with higher values recorded in 2022 than in 2023. The cv. Overture showed higher values in both years as compared to cv. Andreia (**Table 1**). Thus, in 2022, FAN values ranged from 129.78 mg L^-1^ to 134.67 mg L^-1^, whereas in 2023, the FAN values were lower, ranging from 116.78 mg L^-1^ to 124.65 mg L^-1^ (**Figure 5A**). Regarding ST, no significant differences were observed across genotypes and treatments in both years, with lower ST values for cv. Overture in intermediate for both years (**Figure 5B**). As for FT, in 2022, a significant interaction between genotypes and treatments was detected (p = 0.0130), indicating differential behaviour in cv. Andreia among treatments, whereas a higher stability was observed in cv. Overture (**Figure 5C**). Thus, for cv. Andreia, intermediate and late treatments showed lower FT than the control (< 60 min), whereas early treatment showed higher ST (> 60 min) (**Figure 5C**). In 2023, a similar trend was observed in both genotypes and treatments, although only significant differences were reported between genotypes (p = 0.0179) (**Table 4**). In summary, cv. Overture showed a higher stability, without differences among treatments, whereas cv. Andreia had a higher variability (**Figure 5C**).

### 3.5 Multivariate relationships and response patterns

Considering both years, several significant correlations were found among the variables evaluated (p < 0.0001) (**Supplementary Figure 1**). As expected, disease parameters (DI and DS) were strongly positively associated (r_s_ = 0.92). Moreover, both disease parameters were significantly correlated with thin fraction grains (SP2.2), although the correlations were weak (r_s_ = 0.32 and r_s_ = 0.33, respectively). Regarding FDK, significant negative correlations with industrial malting parameters (PS, KI, and WpH) were detected, with weak to moderate correlations (r_s_ = -0.43, r_s_ = -0.44, and r_s_ = -0.39, respectively). As for asymptomatic grains (BTDF), a negative correlation with ST was observed, with a weak correlation (r_s_ = -0.34). The GN were highly correlated with GY (r_s_ = 0.98), whereas no correlation was observed between GW and GY. Regarding HW, significant positive (HW, PC, MP) and negative (ME and KI) correlations were detected, showing moderate correlations depending on the case (r_s_ = 0.48, r_s_ = 0.56, r_s_ = 0.54, r_s_ = -0.44, and r_s_ = -0.59, respectively) (**Supplementary Figure 1**). As for commercial parameters, a significant negative correlation (r_s_ = -0.71) between PG and WS was observed, whereas a moderate positive correlation between PG and MF (r_s_ = 0.50) was observed. Regarding SP2.5, similar correlations to HW were observed, with moderate positive correlations for PC and MP (r_s_ = 0.53 for both), whereas weak to moderate negative correlations were observed for ME and KI (r_s_ = -0.36 and r_s_ = -0.56, respectively). The WS was moderately and positively correlated with WpH and KI (r_s_ = 0.71 and r_s_ = 0.61, respectively), whereas a negative correlation was observed for MP (r_s_ = -0.63). Regarding MF, a strong positive correlation was observed with FAN values (r_s_ = 0.98), for ME, this parameter was moderately and positively correlated with KI (r_s_ = 0.67) and MP (r_s_ = -0.69). Finally, WpH was moderately and negatively associated with MP (r_s_ = -0.76), whereas MP and KI were strongly negatively correlated (r_s_ = -0.96) (**Supplementary Figure 1**).

Regarding the PCA analysis, in 2022, the first principal component primarily captured variation (15.55%) associated with grain quality and nitrogen–related traits, represented mainly by variables such as MP, KI, PC, MF, FAN, DI, and GP (**Supplementary Figures 2A, B**). The second component (28.29%) separated malt quality traits (ME and FT) and yield–related traits (GN, GW, GY) from indicators of disease severity (GP, FDK). Higher–order components (PC3 and PC4) were dominated by variables such as SP2.2 and ST, representing more specific biochemical fractions and secondary grain attributes, whereas PC5 captured residual patterns in grain quality and damage. In 2023, the multivariate structure was more clearly differentiated, with the first principal component representing a strong gradient (18.39%) of grain composition and nitrogen metabolism (MPC, KI, PC, FAN, MF, and ME) (**Supplementary Figures 2B, C**). The second component reflected variation (29.67%) in physical grain quality traits such as GP, HW, FT, and WS. In contrast, disease–related variables (DI, DS, BTDF) loaded heavily and almost exclusively on the third component, whereas higher components (PC4 and PC5) captured specialized biochemical parameters (ST, SP2.2) and pH–related variability (WpH), respectively.Principio del formulario.

Regarding application time, several variables were significantly associated with relative changes (%) compared with the control, including disease variables (DI and DS) and industrial malting parameters (FT and WpH) (**Supplementary Figure 3**). A weak but statistically significant negative linear relationship was observed between DI and the timing of elicitor application (p = 0.037, R² = 0.103), with DI decreasing by approximately 2.5 units for each increment in application stage. Similarly, a weak but statistically significant negative linear relationship was observed between DS and the timing of elicitor application (p = 0.040, R² = 0.095), with DS decreasing by approximately 1.5 units for each increment in application stage. For FT, a weak but statistically significant negative linear relationship was observed between too, (p = 0.016, R² = 0.132), with FT decreasing by approximately 12.5 units for each increment in application stage; whereas for a WpH a weak but statistically significant positive linear relationship was observed between WpH (p = 0.027, R² = 0.096), with WpH increasing by approximately 0.017 units for each increment in application stage. Moreover, several grain and malt quality variables (ME, MF, KI, FAN, MP, PC, and SP2.5) exhibited consistent directional patterns with respect to the phenological timing of elicitor application; however, these relationships were not statistically significant. Interestingly, parameters such as ME, MF, KI, and FAN showed negative trends with the timing of PSP1 application, whereas PC, MP, and SP2.5 showed positive trends along the same application gradient (**Supplementary Figure 3**).

## 4 Discussion

The present work evaluated the impact of a defence elicitor (PSP1) applied under field conditions in two–row barley genotypes over two consecutive growing seasons in the Argentinean pampas. An integrated approach was adopted, encompassing disease parameters, grain yield, commercial and industrial quality. Climatic conditions differed between the seasons evaluated in our work (2022 and 2023), indicating a low–moderate FHB disease pressure. Although conditions during the 2023 susceptibility period were more favourable for FHB (“El Niño ENSO phase and higher rainfalls), incidence and severity were not increased and were even higher in 2022 (“La Niña ENSO” phase, drier conditions). In this case, the observed patterns may point to a temperature–driven response in barley rather than humidity, as mean temperatures in 2022 were higher than historical records, whereas humidity and rainfall were greater in 2023. Similarly, in previous field studies in the Argentine Pampas, weak relationships with key environmental drivers, particularly relative humidity, rainfall, and temperature, have been reported in barley–*Fusarium* interaction studies (Manno et al., 2024; Martínez et al., 2020; Nogueira et al., 2018). Under artificial inoculation experiments, low–moderate FHB pressures were previously found in five barley spring genotypes (two–row), showing incidence values below 20% except for a growing season strongly influenced by “El Niño ENSO phase” in which values were higher (ca. 60%) (Martínez et al., 2020). Moreover, other studies conducted in Germany and Italy indicate that FHB infection in malting barley may be mainly temperature–driven, with high average and maximum temperatures during heading as the most relevant parameters (Beccari et al., 2017; Linkmeyer et al., 2016). These evidences suggest a close relationship between FHB development in barley and climatic interannual variability, with additional sources of variation including genotype–specific resistance mechanisms, genotype–dependent flowering patterns, and unfavourable conditions during disease progression or grain filling (Hoheneder et al., 2022; Page et al., 2025; Schöneberg et al., 2018). Flowering traits may also contribute, since barley exhibits limited anther extrusion and early pollination during heading compared with other cereals such as rye, triticale, and wheat (which usually occur at DC.65) (Alqudah and Schnurbusch, 2017).

Previous studies have confirmed the efficacy of PSP1 as a plant defence elicitor, showing its potential against fungal diseases in strawberry (*Colletotrichum*) and soybean (*Cercospora and Septoria*) (Chalfoun et al., 2018). However, the data presented here do not confirm these defense priming effects in barley under field conditions, showing erratic response patterns and suggesting a better performance of medium–late applications (from stem elongation to heading), close to the crop infection susceptibility period, than early treatments (tillering). It is known that several elicitors are effective against fungal diseases, with distinct mechanisms of action and triggering different plant responses in several agronomic crops (De Vega et al., 2021; Lavanya et al., 2022; Tamm et al., 2011; Thakur and Sohal, 2013). In the case of PSP1, this elicitor induces oxidative signalling, phenolic accumulation, cell wall reinforcement, salicylic acid accumulation, and activation of SAR–related defence genes (Chalfoun, 2009). Thus, the limited reduction in FHB symptoms and disease parameters observed in our work could be attributed to a combination of factors, including: (i) the low–moderate FHB pressure explored and unfavourable conditions for a large–scale FHB outbreak; (ii) the narrow temporal window for effective elicitor–mediated priming; and (iii) the hemibiotrophic infection strategy of *F. graminearum*, in which an initial biotrophic phase is rapidly followed by a necrotrophic phase that is less responsive to salicylic acid–mediated and SAR–related defence pathways induced by PSP1.

Regarding grain yield and its ecophysiological components, no statistically significant effects of elicitor application were detected under the field conditions evaluated in this study. Nevertheless, a consistent trend was observed whereby early applications (tillering) tended to favour grain number and overall grain yield. In contrast, later applications (heading) were associated with slightly higher grain weight and hectolitre weight, traits that appeared comparatively more stable across treatments. This pattern suggests a phenology–dependent modulation of yield components rather than a uniform yield response. Similar results were reported in a previous multi–year field study (2019–2022) evaluating PSP1 in wheat, in which no significant effects on grain weight or grain yield were observed (Martínez et al., 2024), reinforcing the notion that elicitor–driven yield responses are not systematic across all production scenarios.

Despite the absence of significant yield gains in the present study, extensive evidence supports the potential of biostimulants (both microbial and non–microbial) and plant defence elicitors to improve cereal performance under field conditions, including grain yield, abiotic stress tolerance, and quality–related traits (Haggag Wafaa et al., 2017; Różyło et al., 2021; Schütz et al., 2018). A global meta–analysis reported an average increase in grain yield of 17.9% for non–microbial biostimulants and comparable gains (~ 20%) for microbial products, particularly under arid and semi–arid conditions (Li et al., 2022; Schütz et al., 2018). These responses are strongly modulated by environmental and management factors, with greater benefits consistently observed in marginal and low–yield potential systems (Li et al., 2022). In barley, however, field–based evidence on elicitor effects remains scarce and is mainly derived from broader biostimulant studies. A long–term experiment in Uruguay (2009–2023) showed strong grain yield responses to humic–acid–based biostimulants under low–productivity conditions (Izquierdo et al., 2025), underscoring the role of environmental constraints. Mechanistically, elicitors can enhance yield indirectly by priming stress–response pathways related to gene expression, hormonal signalling, and redox regulation (Drobek et al., 2019; Geelen and Xu, 2020; Sharma et al., 2019). However, under fertile soils and humid conditions such as those prevailing in the present study (~1200 mm annually), these priming effects are likely to remain unexpressed, providing a plausible explanation for the lack of measurable yield responses and aligning with reports of reduced biostimulant efficacy in low–stress, high–input environments (Li et al., 2022).

Protein content (9–12%), germinative capacity (> 95%), and grain size (> 2.5 mm) are the main relevant commercial parameters worldwide for malting barley, usually being the primary focus for farmers and plant breeders (Miralles et al., 2020; Kumar et al., 2013). Regarding industrial parameters, malt extract is probably the most critical parameter for maltsters, homebrewers, and breweries, although malt friability, FAN, Kolbach index, and wort pH, among other factors, are also relevant for the malting industry (Kumar et al., 2022a). In the present study, all these commercial and industrial parameters were evaluated according to international EBC protocols, although other essential parameters, such as diastatic power, β–glucans, wort viscosity, and wort colour, were not evaluated in the present study.

It is well established that biostimulants and plant defence elicitors can induce or modulate changes in crop functional properties (Aloo et al., 2023; Biszczak et al., 2025; Różyło et al., 2021). For instance, in wheat cultivated in the study area, recent reports indicate significant effects on grain quality and rheological properties following biostimulant application under field conditions (Arrigoni et al., 2025). In the case of humic substances (HS), they have been shown to promote yield gains through direct biostimulation, including improved nutrient availability and uptake, stimulation of plant growth pathways, and mimicking plant hormones such as auxins, which stimulate root growth and boost metabolic processes such as photosynthesis and respiration (Maffia et al., 2025). However, the underlying mechanisms and their consistency across environments remain incompletely understood in cereals such as barley. Our results suggest an association between FHB disease parameters and malting quality attributes, with the strength of this relationship strongly influenced by the timing of PSP1 application. Early applications were associated with improved malting quality, whereas late applications slightly reduced FHB impact. Conversely, treatments that minimized disease severity were often associated with less favourable malting–quality responses, suggesting a timing–dependent response between defence activation and grain–quality formation.

In our work, correlation patterns indicated that disease parameters were weakly associated with grain size (thin grains) but more consistently linked to impaired endosperm integrity and proteolytic modification, as reflected by negative associations between symptomatic and asymptomatic grains and soluble protein, Kolbach index, wort pH, and saccharification time, suggesting endosperm deterioration. Thus, multivariate analyses highlighted multifactorial relationships among *Fusarium* expression, grain structure, and malting quality, in accordance with previous studies (Schwarz et al., 2006; Kumar et al., 2022b; Nielsen et al., 2014). The PCA showed year–dependent patterns, with a productivity–disease relationship in 2022 and an independent *Fusarium* infection axis in 2023, consistent with climate–driven decoupling between disease severity and yield or quality traits. In agreement, several previous studies have reported a climate–driven decoupling between FHB development and yield penalties or quality traits (Doohan et al., 2003; Linkmeyer et al., 2016; Schöneberg et al., 2018). As to monotonic analyses, when application time was treated as an ordinal phenological factor, some statistically significant but overall weak associations were observed, suggesting that timing alone may explain only a limited portion of the observed variability. In line with this, previous evidence in wheat treated with PSP1 under field conditions suggested defence–related energetic costs that affect grain composition rather than grain yield or disease outcomes (Martínez et al., 2024).

Overall, the contrasting effects of PSP1 on FHB suppression and malting quality indicate a relationship that depends on application timing within the barley–*F. graminearum* interaction, reflecting underlying physiological and biochemical processes during the crop cycle and grain development. Applications close to heading resulted in a slight reduction in FHB, consistent with salicylic acid–mediated defence priming occurring during the initial biotrophic phase of *Fusarium* infection, which typically spans the first 24–84 h after host colonization, and fitting within the growth–defence balance framework (Giedrojć et al., 2025; Huot et al., 2014). However, elicitor application at this stage may plausibly alter carbon and nitrogen allocation dynamics, elevating metabolic costs and thereby constraining starch and protein accumulation during grain set and the early grain–filling phase (Bolton, 2009; Heil and Baldwin, 2002; Huot et al., 2014; Miralles et al., 2011). Such shifts may alter the balance and composition of grain reserves, leading to changes in key malting parameters, including increased grain protein concentration, altered protein–to–starch ratios and grain size, which can ultimately reduce malt friability and extract, as observed in this study. These responses could indicate reduced endosperm availability and impaired overall malt modification, although malt modification per se was not directly quantified (Briggs, 1998; Bolton, 2009).

In addition, defence–induced stress responses may affect grain protein composition rather than total protein content, favouring the accumulation of defence–related proteins with lower proteolytic accessibility during malting, thereby negatively impacting FAN release and the Kolbach index (Gupta et al., 2010). Defence priming, involving salicylic acid, jasmonic acid and ethylene, may further antagonize growth–promoting hormones such as gibberellins, potentially interfering with endosperm modification, enzymatic potential and biochemical maturation under low–stress, high–input conditions (Briggs, 1998; Robert–Seilaniantz et al., 2011; Li et al., 2022). Thus, plant hormonal networks can constitute a finely tuned regulatory system that balances growth with adaptation to abiotic stress. One of the most studied plant hormones is auxin, which plays a key role in plant growth and influences cell elongation, division, and differentiation. When plants face abiotic stress, auxin production, transport, and signaling change, thereby affecting root structure and stress responses (Jing et al., 2023). Although potential effects on hordein fractions were not assessed in the present study, they warrant further investigation, particularly given the susceptibility of hordeins to protease degradation by *Fusarium* species (Pekkarinen et al., 2003; Sarlin et al., 2005). Collectively, these findings underscore a reduced FHB mitigation achieved through PSP1 application near heading may come at the expense of barley technological quality, highlighting the critical importance of application timing when deploying elicitors in malting–oriented production systems.

## 5 Conclusion

The results presented here provide a novel field–based perspective on plant–pathogen interactions, illustrating how a plant defence elicitor can modulate disease expression alongside key agronomic and malting–quality traits. Under the field conditions evaluated, PSP1 application in two–row barley did not result in consistent or statistically significant reductions in FHB or grain yield. However, a clear time–dependent response emerged, revealing a relationship between disease mitigation and malt quality. Although treatment effects were generally minor, consistent directional responses were observed across the climatic conditions explored. Applications made close to heading were associated with low reductions in FHB incidence and severity but negatively affected key malting parameters. A hypothetical explanation for these observed patterns could be related to the dynamics of starch and protein deposition during grain set and early grain filling. However, direct physiological measurements were beyond the scope of this study and could be approached in future experiments. In contrast, earlier applications (from tillering to stem elongation) showed more stable or favourable malting quality profiles but provided limited disease suppression.

**Figure 6:**
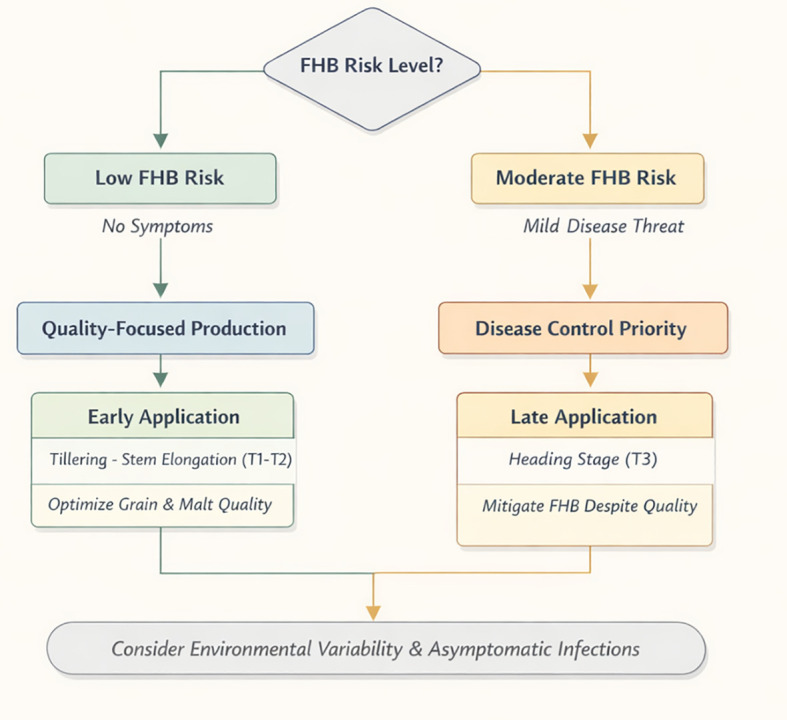
Flowchart showing the PSP1 application decision guide based on the evaluated FHB pressure and the reported results.

Taken together, these results indicate that the timing of PSP1 application is a critical determinant of its agronomic and technological outcomes in barley. The results presented define a relationship between quality preservation and disease mitigation that can inform decision–making under contrasting production scenarios. Based on these findings, PSP1 use could be framed within a simple decision context: early applications may be prioritized under quality–driven systems, whereas late applications could be considered under low–moderate FHB risk (**Figure 6**). However, under high FHB pressure, the effects of late applications near heading should be interpreted with caution, as they are likely specific to low-to-moderate disease pressure and may differ under more severe epidemic conditions. Evidence on the use of defence elicitors in barley remains scarce, underscoring the need for further multi–environment and stress–gradient studies to better define the conditions under which elicitor–based strategies translate into consistent disease control and yield benefits. A potential integrated FHB management approach remains dependent on environmental conditions and the inherent difficulty of accurately assessing FHB in barley crops under field conditions, particularly given the high number of asymptomatic grains. Therefore, PSP1 should be integrated as a context–dependent tool, with timing decisions aligned with disease pressure, complemented by genetic resistance and traditional fungicides, and considering environmental conditions and end–use quality objectives, rather than as a uniform disease–control strategy.

## Data Availability

The raw data supporting the conclusions of this article will be made available by the authors, without undue reservation.
