# Supplementary material for: Timing–dependent responses of Fusarium graminearum suppression and malting quality in barley following PSP1 elicitor application
Source: Front Plant Sci. 2026 May 12;17:1838206. doi: 10.3389/fpls.2026.1838206 (PMC13201413; doi:10.3389/fpls.2026.1838206)
Supplement: Supplementary file 1 [file DataSheet1.docx]

Supplementary Material

## Supplementary Figures

**
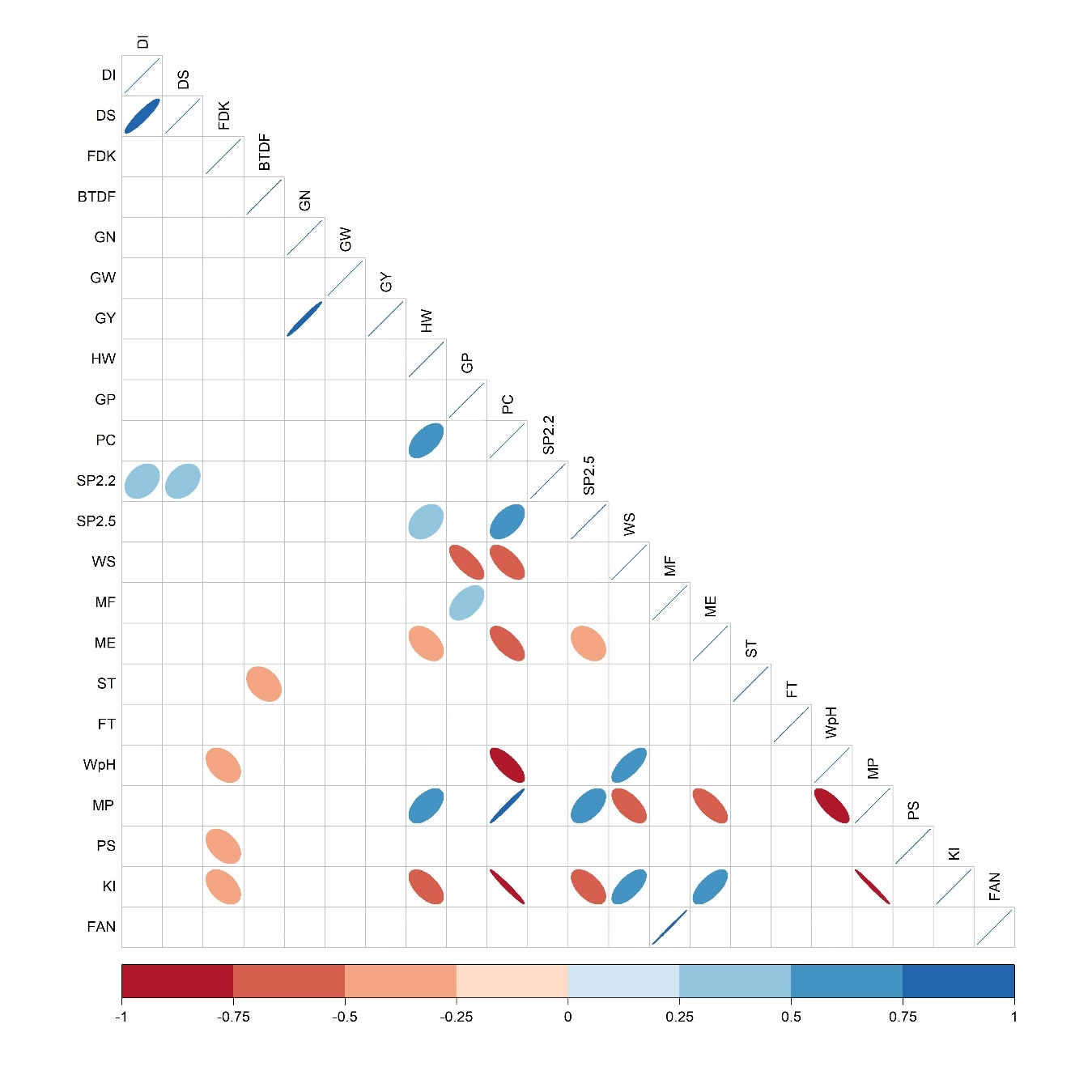
**

**Supplemental Figure 1.** Correlogram of the analysed variables based on Spearman's rank correlation coefficients (r_s_). The colour scale denotes the direction and magnitude of the correlations, with red (●) indicating negative associations and blue (●) indicating positive associations.

**
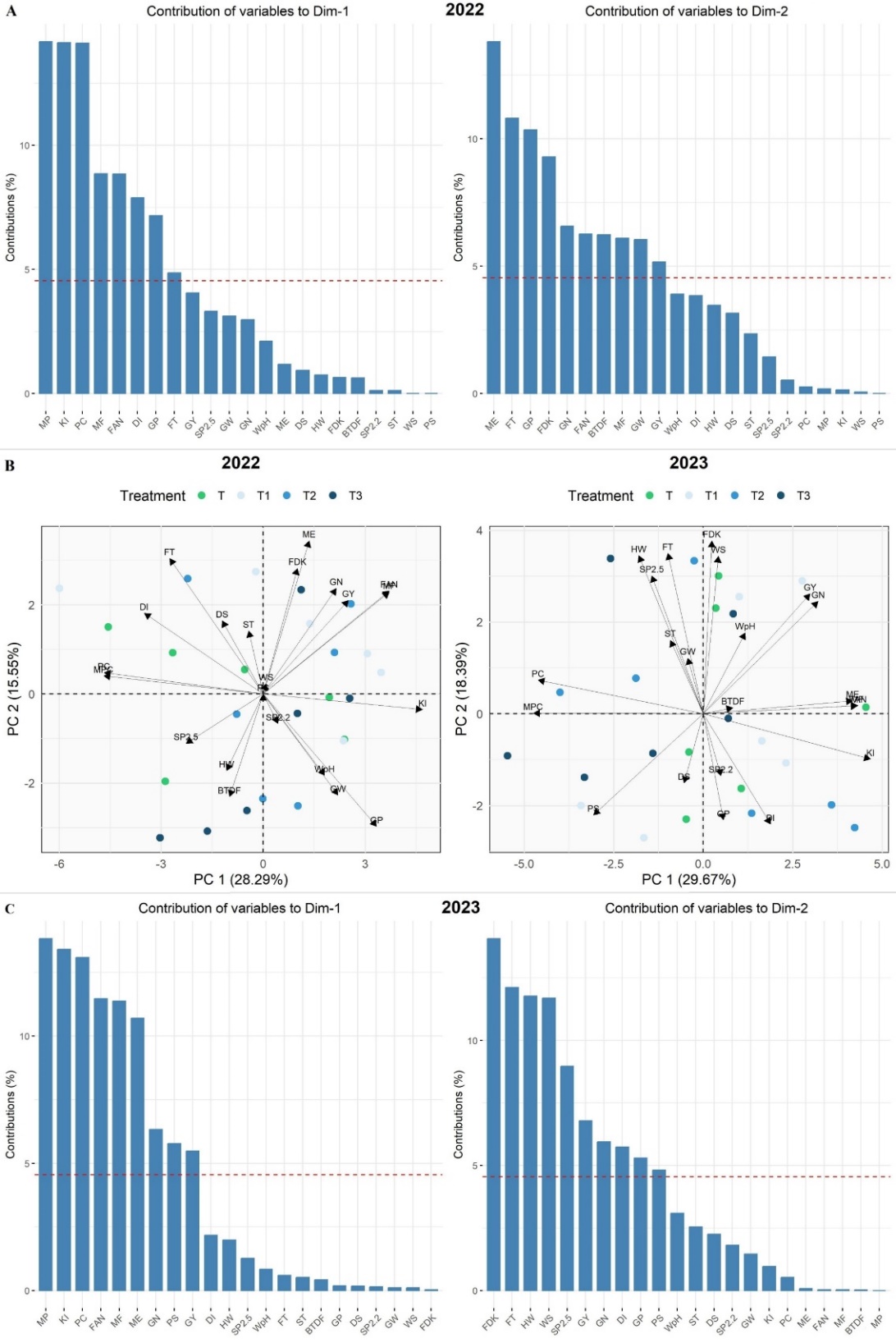
**

**Supplemental Figure 2.** Principal component analysis (PCA) and scree plots, partitioned by year (2022 and 2023) and genotypes (cv. Andreia and cv. Overture). A) Scree plot for 2022. Left: dimension one (PC1). Right: dimension two (PC2). B) PCA for 2022 (left) and 2023 (right). C) Scree plot for 2023. Left: dimension one (PC1). Right: dimension two (PC2).

**
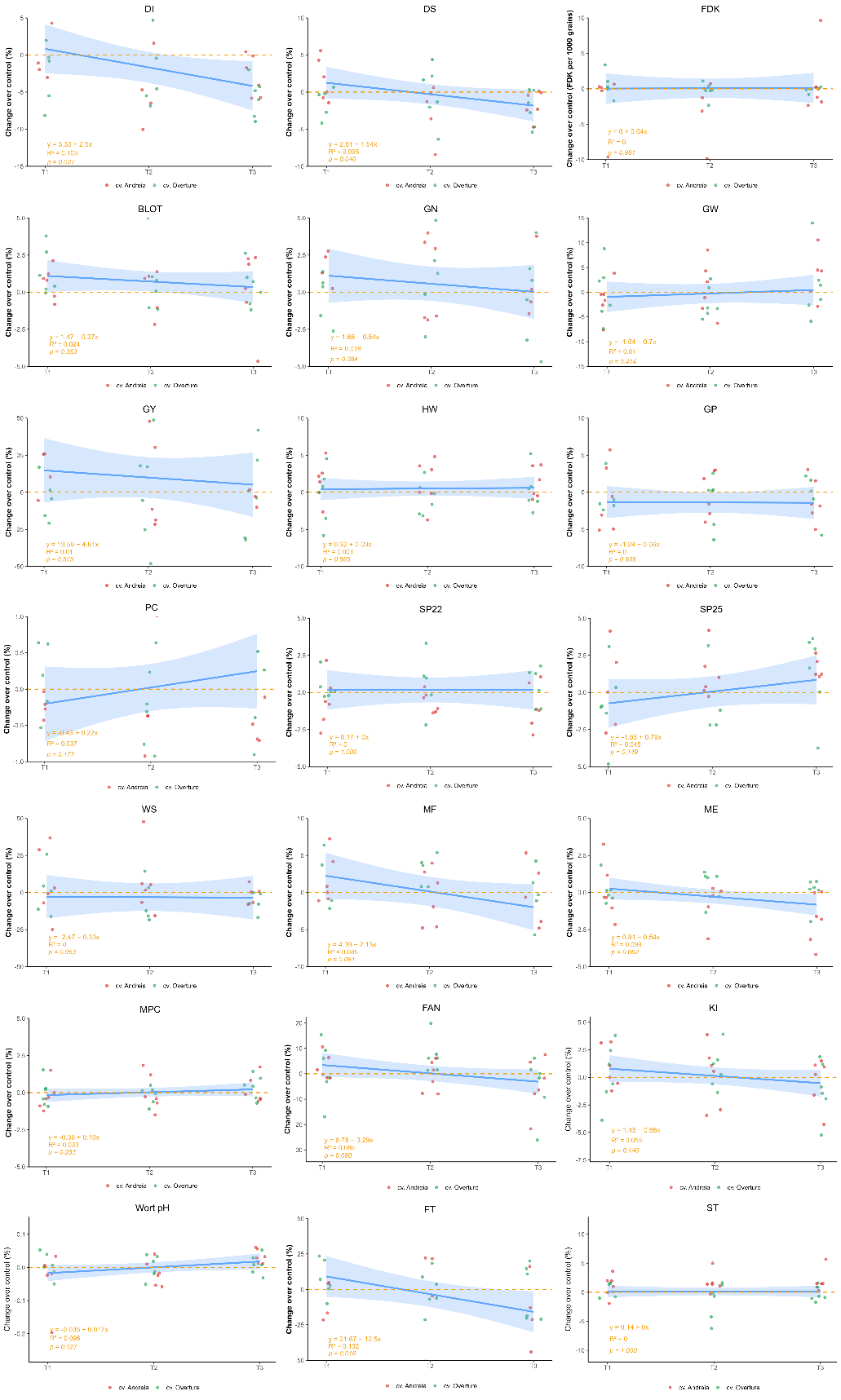
**

**Supplemental Figure 3.** Monotonic relationships between the evaluated variables (expressed as percentage change relative to the control) and PSP1 application timing (y–axis). Treatments correspond to PSP1 applied at tillering (DC 21, T1), stem elongation (DC 31, T2), and 50% heading (DC 55, T3).

**Supplemental Tables**

**Supplemental Table 1.** Estimated marginal means (EMMs) with 95% confidence intervals (CI95) for each disease parameter evaluated. DI: disease incidence. DS: disease severity. FDK: Fusarium damaged-kernel. BLOT: blotter test deep freezing.

| **Year** | **Genotype** | **n** | **DI** | |  | **DS** | |  | **FDK** | | | **BLOT** | |
| --- | --- | --- | --- | --- | --- | --- | --- | --- | --- | --- | --- | --- | --- |
|  | **&Treatment** |  | EMMs ± SE | CI95 |  | EMMs ± SE | CI95 |  | EMMs ± SE | CI95 |  | EMMs ± SE | CI95 |
|  | cv. Andreia | 12 | 7.62% ± 2.1% | [4.4%, 12.9%] |  | 4.36% ± 1.23% | [2.49%, 7.51%] |  | 0.26% ± 0.04% | [0.18%, 0.38%] |  | 2.13% ± 0.42% | [1.45%, 3.13%] |
|  | cv. Overture | 12 | 4.29% ± 1.6% | [2.05%, 8.79%] |  | 0% | n.e. |  | 0.26% ± 0.04% | [0.19%, 0.37%] |  | 1.95% ± 0.41% | [1.3%, 2.93%] |
| 2022 | T | 6 | 7.24% ± 2.59% | [3.53%, 14.27%] |  | 3.4% ± 1.53% | [1.4%, 8.05%] |  | 0.30% ± 0.01% | [0.19%, 0.47%] |  | 1.49% ± 0.50% | [0.77%, 2.85%] |
|  | T1 | 6 | 6.59% ± 2.70% | [2.9%, 14.3%] |  | 2.8% ± 1.54% | [0.94%, 8.02%] |  | 0.35% ± 0.01% | [0.23%, 0.54%] |  | 2.33% ± 0.62% | [1.39%, 3.90%] |
|  | T2 | 6 | 7.95% ± 2.75% | [3.97%, 15.27%] |  | 3.94% ± 1.67% | [1.7%, 8.87%] |  | 0.23% ± 0.01% | [0.14%, 0.39%] |  | 2.00% ± 0.57% | [1.14%, 3.49%] |
|  | T3 | 6 | 2.8% ± 1.65% | [0.87%, 8.66%] |  | 0% | n.e. |  | 0.20% ± 0.01% | [0.11%, 0.35%] |  | 2.49% ± 0.64% | [1.51%, 4.10%] |
|  | cv. Andreia | 12 | 0% | n.e. |  | 0% | n.e. |  | 0.14% ± 0.18% | [0.04%, 1.81%] |  | 1.70% ± 0.44% | [1.02%, 2.82%] |
|  | cv. Overture | 12 | 7.2% ± 1.78% | [4.4%, 11.55%] |  | 3.27% ± 1.14% | [1.64%, 6.42%] |  | 0% | n.e. |  | 1.50% ± 0.39% | [0.90%, 2.51%] |
| 2023 | T | 6 | 7.1% ± 2.42% | [3.6%, 13.55%] |  | 2.41% ± 0.13% | [0.78%, 7.21%] |  | 0% | n.e. |  | 1.27% ± 0.48% | [0.61%, 2.65%] |
|  | T1 | 6 | 7.7% ± 2.54% | [3.97%, 14.4%] |  | 3.14% ± 1.69% | [1.08%, 8.77%] |  | 0% | n.e. |  | 2.46% ± 0.68% | [1.43%, 4.20%] |
|  | T2 | 6 | 0% | n.e. |  | 0% | n.e. |  | 0% | n.e. |  | 2.55% ± 0.70% | [1.49%, 4.34%] |
|  | T3 | 6 | 3.33% ± 1.64% | [1.26%, 8.54%] |  | 1.39% ± 0.98% | [0.35%, 5.30%] |  | 0% | n.e. |  | 0.81% ± 0.37% | [0.32%, 2.00%] |

*n.e.: not estimable.

**Supplemental Table 2.** Estimated marginal means (EMMs) with 95% confidence intervals (CI95) for each grain yield parameter evaluated. GN: grain number. GW: grain weight. GY: grain yield. HW: hectoliter weight.

| **Year** | **Genotype** | **n** | **GN** | | **GW** | | |  | **GY** | | | | **HW** | |
| --- | --- | --- | --- | --- | --- | --- | --- | --- | --- | --- | --- | --- | --- | --- |
|  | **&Treatment** |  | EMMs ± SE | CI95 |  | EMMs ± SE | CI95 |  | EMMs ± SE | CI95 |  | EMMs ± SE | | CI95 |
|  | cv. Andreia | 12 | 11 528 ± 557 | [10 209, 12 846] |  | 47.46 ± 0.32 | [46.68, 48.23] |  | 546.54 ± 25.87 | [485.36, 607.73] |  | 64.80 ± 0.45 | | [63.64, 65.95] |
|  | cv. Overture | 12 | 11 487± 557 | [10 168, 12 805] |  | 48.11 ± 0.32 | [47.33, 48.88] |  | 552.31 ± 25.88 | [491.12, 613.49] |  | 64.36 ± 0.46 | | [63.21, 65.51] |
| 2022 | T | 6 | 10 074 ± 788 | [8 383, 11 765] |  | 47.98 ± 0.45 | [47.01, 48.95] |  | 483.45 ± 36.59 | [404.97, 561.93] |  | 64.48 ± 0.61 | | [63.15, 65.81] |
|  | T1 | 6 | 12 656 ± 788 | [10 965, 14 347] |  | 47.72 ± 0.45 | [46.74, 48.69] |  | 604.08 ± 36.59 | [525.60, 682.56] |  | 64.33 ± 0.61 | | [63.00, 65.66] |
|  | T2 | 6 | 11 506 ± 788 | [9 815, 13 197] |  | 47.82 ± 0.45 | [46.84, 48.79] |  | 550.13± 36.60 | [471.64, 628.61] |  | 65.10 ± 0.61 | | [63.77, 66.43] |
|  | T3 | 6 | 11 793 ± 788 | [10 102, 13 484] |  | 47.62 ± 0.45 | [46.64, 48.59] |  | 560.05± 36.60 | [481.56, 638.53] |  | 64.40 ± 0.61 | | [63.07, 65.73] |
|  | cv. Andreia | 12 | 9 187 ± 793 | [7 310, 11 064] |  | 47.53 ± 0.69 | [45.89, 49.17] |  | 439.08± 38.61 | [347.78, 530.39] |  | 63.68 ± 0.33 | | [62.89, 64.47] |
|  | cv. Overture | 12 | 9 315± 793 | [7 439, 11 192] |  | 48.09 ± 0.69 | [46.45, 49.73] |  | 446.42 ± 38.61 | [355.12, 537.73] |  | 61.93 ± 0.33 | | [61.14, 62.72] |
| 2023 | T | 6 | 9 827± 1122 | [7 420, 12 235] |  | 47.95 ± 0.98 | [45.85, 50.05] |  | 472.53 ± 54.61 | [355.41, 589.65] |  | 62.45 ± 0.47 | | [61.45, 63.45] |
|  | T1 | 6 | 9 628 ± 1122 | [7 221, 12 035] |  | 47.35 ± 0.98 | [45.25, 49.45] |  | 455.08 ± 54.61 | [337.96, 572.20] |  | 63.22 ± 0.47 | | [62.22, 64.22] |
|  | T2 | 6 | 9 234± 1122 | [6 826, 11 641] |  | 47.3 ± 0.98 | [45.20, 49.40] |  | 436.13 ± 54.61 | [319.01, 553.25] |  | 62.12 ± 0.47 | | [61.12, 63.11] |
|  | T3 | 6 | 8 315± 1122 | [5 908, 10 723] |  | 48.65 ± 0.98 | [46.55, 50.75] |  | 407.28 ± 54.61 | [290.15, 524.39] |  | 63.42 ± 0.47 | | [62.42, 64.42] |

**Supplemental Table 3.** Estimated marginal means (EMMs) with 95% confidence intervals (CI95) for each commercial parameter evaluated. GP: germinative power. PC: protein content. SP2.2: screening percentage <2.2 mm. SP2.5: screening percentage >2.5 mm. WS: water sensitivity.

| **Year** | **Genotype** | **n** | **GP (%)** | | **PC (%)** | | **SP2.2 (%)** | | | **SP2.5 (%)** | | **WS (%)** |
| --- | --- | --- | --- | --- | --- | --- | --- | --- | --- | --- | --- | --- |
|  | **&Treatment** |  | EMMs ± SE | CI95 | EMMs ± SE | CI95 | EMMs ± SE | CI95 | EMMs ± SE | CI95 | EMMs ± SE | CI95 |
|  | cv. Andreia | 12 | 85.11 ± 0.98 | [83.07, 86.93] | 13.80 ± 0.19 | [13.24, 14.36] | 2.85 ± 0.49 | [2.03, 3.97] | 92.87 ± 0.74 | [91.26, 94.20] | 3.42 ± 1.36 | [0.21, 6.62] |
|  | cv. Overture | 12 | 85.59 ± 0.95 | [83.61, 87.36] | 13.05 ± 0.19 | [12.45, 13.61] | 3.59 ± 0.56 | [2.64, 4.86] | 91.58 ± 0.81 | [89.86, 93.03] | 4.00 ± 1.36 | [0.79, 7.21] |
| 2022 | T | 6 | 86.19 ± 1.34 | [83.35, 88.61] | 13.78 ± 0.24 | [13.23, 14.34] | 3.06 ± 0.72 | [1.93, 4.82] | 91.34 ± 1.15 | [88.81, 93.34] | 5.33 ± 1.92 | [1.22, 9.45] |
|  | T1 | 6 | 84.68 ± 1.40 | [81.73, 87.22] | 13.23 ± 0.24 | [12.68, 13.79] | 2.65 ± 0.66 | [1.62, 4.29] | 92.02 ± 1.12 | [89.53, 93.96] | 2.67 ± 1.92 | [-1.45, 6.78] |
|  | T2 | 6 | 85.04 ± 1.38 | [82.14, 87.55] | 13.25 ± 0.24 | [12.69, 13.81] | 4.60 ± 0.89 | [3.14, 6.71] | 92.18 ± 1.10 | [89.74, 94.08] | 5.50 ± 1.92 | [1.38, 9.62] |
|  | T3 | 6 | 85.45 ± 1.36 | [82.58, 87.92] | 13.43 ± 0.24 | [12.88, 13.99] | 2.79 ± 0.68 | [1.73, 4.47] | 93.33 ± 1.02 | [91.04, 95.07] | 1.33 ± 1.92 | [-2.78, 5.45] |
|  | cv. Andreia | 12 | 62.30 ± 1.21 | [59.90, 64.65] | 11.69 ± 0.22 | [11.11, 12.28] | 3.00 ± 0.49 | [2.17, 4.13] | 91.25 ± 0.82 | [89.50, 92.74] | 47.17 ± 5.44 | [34.31, 60.03] |
|  | cv. Overture | 12 | 63.90 ± 1.18 | [61.55, 66.19] | 10.59 ± 0.22 | [10.01, 11.18] | 3.33 ± 0.52 | [2.45, 4.51] | 88.11 ± 0.94 | [86.15, 89.83] | 26.67 ± 5.44 | [13.81, 39.53] |
| 2023 | T | 6 | 64.15 ± 1.69 | [60.78, 67.38] | 10.75 ± 0.29 | [10.11, 11.39] | 3.16 ± 0.71 | [2.02, 4.91] | 90.46 ± 1.21 | [87.82, 92.58] | 40.00 ± 7.69 | [23.50, 56.50] |
|  | T1 | 6 | 62.63 ± 0.17 | [59.24, 65.89] | 11.08 ± 0.29 | [10.44, 11.73] | 3.16 ± 0.71 | [2.02, 4.91] | 88.17 ± 1.32 | [85.33, 90.52] | 38.17 ± 7.69 | [21.67, 54.66] |
|  | T2 | 6 | 64.05 ± 1.68 | [60.69, 67.27] | 10.95 ± 0.29 | [10.31, 11.59] | 3.32 ± 0.73 | [2.15, 5.10] | 90.51 ± 1.23 | [87.80, 92.67] | 31.33 ± 7.69 | [14.84, 47.83] |
|  | T3 | 6 | 61.58 ± 0.17 | [58.19, 64.86] | 11.78 ± 0.29 | [11.14, 12.43] | 3.00 ± 0.7 | [1.90, 4.71] | 89.86 ± 1.23 | [87.18, 92.04] | 38.17 ± 7.69 | [21.70, 54.66] |

**Supplemental Table 4.** Estimated marginal means (EMMs) with 95% confidence intervals (CI95) for each malting parameter evaluated. MP: malt total protein. MF: malt friability. ME: malt extract. KI: Kolbach index. FAN: free amino nitrogen. WpH: wort pH.

| **Year** | **Genotype** | **n** | **MP** | | | **MF** | | |  | **ME** | | | |
| --- | --- | --- | --- | --- | --- | --- | --- | --- | --- | --- | --- | --- | --- |
|  | **/Treatment** |  | EMMs ± SE | CI95 |  | EMMs ± SE | CI95 | |  | EMMs ± SE | CI95 | |  |
|  | cv. Andreia | 12 | 12.92 ± 0.15 | [12.48, 13.37] |  | 84.28 ± 1.05 | [82.11, 86.24] | |  | 81.58 ± 0.20 | [81.10, 82.07] | |  |
|  | cv. Overture | 12 | 12.33 ± 0.15 | [11.89, 12.78] |  | 85.75 ± 1.01 | [83.65, 87.62] | |  | 81.50 ± 0.20 | [81.01, 81.98] | |  |
| 2022 | T | 6 | 12.92 ± 0.19 | [12.47, 13.37] |  | 83.84 ± 0.15 | [80.67, 86.57] | |  | 81.67 ± 0.29 | [81.05, 82.29] | |  |
|  | T1 | 6 | 12.47 ± 0.19 | [12.02, 12.93] |  | 86.52 ± 0.14 | [83.55, 89.03] | |  | 81.67 ± 0.29 | [81.05, 82.29] | |  |
|  | T2 | 6 | 12.50 ± 0.19 | [12.06, 12.95] |  | 86.21 ± 1.41 | [83.20, 88.75] | |  | 81.67 ± 0.29 | [81.05, 82.29] | |  |
|  | T3 | 6 | 12.63 ± 0.19 | [12.19, 13.08] |  | 83.34 ± 1.52 | [80.14, 86.11] | |  | 81.17 ± 0.29 | [80.55, 81.79] | |  |
|  | cv. Andreia | 12 | 11.16 ± 0.30 | [10.24, 12.08] |  | 78.34 ± 1.19 | [75.91, 80.59] | |  | 82.25 ± 0.46 | [80.90, 83.60] | |  |
|  | cv. Overture | 12 | 10.33 ± 0.30 | [9.41, 11.25] |  | 79.95 ± 1.17 | [77.55, 82.14] | |  | 83.17 ± 0.46 | [81.82, 84.52] | |  |
| 2023 | T | 6 | 10.42 ± 0.36 | [9.55, 11.29] |  | 79.83 ± 1.64 | [76.43, 82.85] | |  | 83.00 ± 0.56 | [81.67, 84.33] | |  |
|  | T1 | 6 | 10.63 ± 0.36 | [9.76, 11.50] |  | 79.86 ± 1.64 | [76.46, 82.88] | |  | 83.33 ± 0.56 | [82.00, 84.66] | |  |
|  | T2 | 6 | 10.68 ± 0.36 | [9.81, 11.55] |  | 81.93 ± 1.60 | [78.59, 84.85] | |  | 82.83 ± 0.56 | [81.50, 84.16] | |  |
|  | T3 | 6 | 11.23 ± 0.36 | [10.36, 12.10] |  | 74.50 ± 1.78 | [70.86, 77.83] | |  | 81.67 ± 0.56 | [80.34, 83.00] | |  |
| **Year** | **Genotype** | **n** | **KI** | | **FAN** | | | | |  | **WpH** | | |
|  | **/Treatment** |  | EMMs ± SE | CI95 |  | EMMs ± SE | | CI95 | |  | EMMs ± SE | CI95 |  |
|  | cv. Andreia | 12 | 33.55 ± 0.40 | [32.39, 34.71] |  | 131.22 ± 1.97 | | [124.99, 137.44] | |  | 6.08 ± 0.01 | [6.06, 6.10] |  |
|  | cv. Overture | 12 | 35.13± 0.40 | [34.00, 36.29] |  | 133.38 ± 1.97 | | [127.16, 139.61] | |  | 6.10 ± 0.01 | [6.08, 6.12] |  |
| 2022 | T | 6 | 33.60± 0.50 | [32.43, 34.76] |  | 130.60 ± 2.31 | | [124.80, 136.40] | |  | 6.07 ± 0.01 | [6.05, 6.10] |  |
|  | T1 | 6 | 34.80 ± 0.50 | [33.64, 35.97] |  | 134.67 ± 2.31 | | [128.87, 140.47] | |  | 6.09± 0.01 | [6.07, 6.11] |  |
|  | T2 | 6 | 34.65 ± 0.50 | [33.49, 35.82] |  | 134.15 ± 2.31 | | [128.35, 139.95] | |  | 6.09 ± 0.01 | [6.07, 6.11] |  |
|  | T3 | 6 | 34.32 ± 0.50 | [33.15, 35.48] |  | 129.78 ± 2.31 | | [123.98, 135.58] | |  | 6.11 ± 0.01 | [6.09, 6.14] |  |
|  | cv. Andreia | 12 | 39.34 ± 0.89 | [36.41, 42.26] |  | 122.15 ± 3.21 | | [111.94, 132.36] | |  | 6.47 ± 0.04 | [6.33, 6.62] |  |
|  | cv. Overture | 12 | 42.40 ± 0.89 | [39.47, 45.32] |  | 124.27 ± 3.21 | | [114.06, 134.48] | |  | 6.44 ± 0.04 | [6.30, 6.59] |  |
| 2023 | T | 6 | 41.42 ± 1.01 | [38.76, 44.07] |  | 124.65 ± 3.76 | | [115.17, 134.14] | |  | 6.48 ± 0.04 | [6.35, 6.60] |  |
|  | T1 | 6 | 41.43 ± 1.01 | [38.77, 44.09] |  | 124.65 ± 3.76 | | [115.17, 134.14] | |  | 6.43 ± 0.04 | [6.30, 6.56] |  |
|  | T2 | 6 | 41.34 ± 1.01 | [38.68, 44.00] |  | 127.15 ± 3.76 | | [117.67, 136.64] | |  | 6.45 ± 0.04 | [6.32, 6.58] |  |
|  | T3 | 6 | 39.28 ± 1.01 | [36.62, 41.94] |  | 116.38 ± 3.76 | | [106.90, 125.87] | |  | 6.48 ± 0.04 | [6.35, 6.61] |  |
